# The Expected Number of Background Disease Events during Mass Immunization in China

**DOI:** 10.1371/journal.pone.0071818

**Published:** 2013-08-20

**Authors:** YouXin Wang, LiJuan Wu, XinWei Yu, FeiFei Zhao, Alyce Russell, ManShu Song, Wei Wang

**Affiliations:** 1 School of Public Health, Capital Medical University, Beijing, China; 2 Municipal Key Laboratory of Clinical Epidemiology, Beijing, China; 3 College of Life Science, University of Chinese Academy of Sciences, Beijing, China; 4 School of Medical Sciences, Edith Cowan University, Perth, Australia; Public Health Ontario, Canada

## Abstract

It is critical to distinguish events that are temporarily associated with, but not caused by, vaccination from those caused by vaccination during mass immunization. We performed a literature search in China National Knowledge Infrastructure and Pubmed databases. The number of coincident events was calculated based on its incidence rate and periods after receipt of a dose of hypothesized vaccine. We included background incidences of Guillain-Barré syndrome, anaphylaxis, seizure, sudden adult death syndrome, sudden cardiac death, spontaneous abortion, and preterm labour or delivery. In a cohort of 10 million individuals, 7.71 cases of Guillain-Barré syndrome would be expected to occur within six weeks of vaccination as coincident background cases. Even for rare events, a large number of events can be expected in a short period because of the large population targeted for immunization. These findings may encourage health authorities to screen the safety of vaccines against unpredictable pathogens.

## Introduction

In 2009, a novel influenza A H1N1 virus emerged from Mexico and spread quickly worldwide. A mass vaccination campaign against this pandemic H1N1 strain was carried out in many countries to cope with this crisis. Up to February 28th 2010, 82.12 million individuals had been vaccinated with pandemic H1N1 influenza vaccines [1]. With such a large population vaccinated, adverse events will inevitably occur and the question will arise as to whether the vaccine is causative. It is critical to distinguish events that are temporarily associated with, but not caused by, vaccination from those caused by vaccination.

Black and colleagues raised the importance of background rates of disease in monitoring H1N1 vaccine safety during mass immunization with pandemic H1N1 influenza vaccines, and estimated the expected background rates of occurrence of several health conditions [2]. Deeks and colleagues estimated background rates of Guillain-Barré Syndrome in Ontario while responding to safety concerns during the pandemic H1N1/09 immunization campaign [3].

## Materials and Methods

In this study, we identified the background rates of selected medical events in China and estimated the safety of the pandemic H1N1 influenza vaccines made in China. The available data would help to distinguish events that are temporarily associated with, but not caused by, vaccination from those caused by vaccination in mass immunization.

The list of possible outcomes that might need to be assessed after receipt of a pandemic H1N1 influenza vaccine was selected by reference to the report of Black et al., 2009 [2]. Guillain-Barré syndrome, anaphylaxis, seizure, sudden adult death syndrome, sudden cardiac death, preterm labour and spontaneous abortion were selected for further analyses. The diagnosis specified for each event was entered as a key word in PubMed, CNKI (China National Knowledge Infrastructure, www.cnki.net) and Wanfang Data (http://www.wanfangdata.com/), and publications that included incidence rates of the diseases were identified. CNKI and Wanfang are the largest and most complete sources of academic information in China. They represent the Chinese equivalents of the Web of Knowledge or PubMed [4], [5]. They include searchable databases with scanned full-text manuscripts from Chinese academic journals, statistical yearbooks, doctoral/masters dissertation theses and proceedings of conferences. CNKI contains more than 25 million research articles, about 100,000 PhD theses, 500,000 Masters Theses and more than 1 million conference papers in Chinese language [6]–[8]. We gave priority to recent publications and to those that included sex-specific and age-specific incidence rates. We also preferred to select publications from active surveillance, and when these were unavailable, the publications from passive surveillance were selected (marked in the text or footnote).

The expected number of coincident events likely to occur within one day, one week, and six weeks were calculated by multiplying the number of hypothetically vaccinated people, the background rates of selected events at selected sites, and the risk periods after receipt of a hypothetical dose of vaccine, by reference to the methods reported in Black et al 2009 [2] and Deeks et al 2011 [3].

## Results

Table 1 summarizes background incidence of selected disease events in China, with age-specific incidence reported when available [9]–[16]. The incidence of Guillain-Barré Syndrome was higher in men than in women, varying from 0.74 to 1.02 per 100,000 person-years in males and 0.57 to 0.79 per 100,000 person-years in females of all ages [9], [10]. The incidence of anaphylaxis was 6.5 per million for attenuated measles vaccine [11]. In Heilongjiang province, the incidence of seizure was 9.9 per 100,000 person-years [12], while in the other provinces, e.g., Henan, Jiangsu, Ningxia and Shanxi, incidence varied from 25.8 to 38.9 per 100,000 person-years [12]. In minority ethnic groups, such as Jinuo in Yunnan province and Tibetan in the Tibet autonomous region, the incidences were 22.4 per 100,000 person-years and 30.0 per 100,000 person-years, respectively [13], [14]. In migrant workers of the cities Dongguan and Shenzhen of Guangdong province, incidence of sudden adult death syndrome reached 110 per 100,000 person-years [15]. Rates of sudden cardiac death (within 1 h of onset of symptoms or in sleep) varied from 19.2 to 56.1 per 100,000 person-years for females and 33.9 to 66.1 per 100,000 person-years for males [16].

**Table 1:** Incidence of disease events that might be temporarily associated with vaccine by genders, provinces and age groups.

Incidence (per 100 000 person-years)	
**Guillain-Barré syndrome**	
Harbin of Heilongjiang [9]	
0–9 years	Female 0.94, male 1.34
10–19 years	Female 0.65, male 0.84
20–29 years	Female 0.72, male 0.50
30–39 years	Female 0.00, male 0.79
40–49 years	Female 1.10, male 0.38
50–59 years	Female 0.00, male 0.89
≥60 years	Female 0.52, male 0.49
All ages*	Female 0.57, male 0.74
Hebei and Beijing [10]	
0–9 years	0.47
10–19 years	0.74
20–29 years	0.70
30–39 years	0.56
40–49 years	1.06
50–59 years	1.43
≥60 years	0.92
All ages¶	
Urban	Female 0.47, male 1.02
Rural	Female 0.79, male 0.92
**Anaphylaxis**	
Sichuan† § [11]	
8 months∼14 years	0.65
**Seizure**	
Heilongjiang [12]	9.9
Henan [12]	32.1
Jiangsu [12]	36.0
Ningxia [12]	25.8
Shanxi [12]	38.9
Yunnan (*Jinuo* ethnic group) [13]	22.4
Lhase of Tibet (*Tibetan* ethnic group) [14]	30.0
**Sudden adult death syndrome**	
Dongguan & Shenzhen of Guangdong§ [15]	
15∼52 years	110
Sudden cardiac death	
Beijing [16]	
≥25years ‡	Female 19.2, male 37.3
Guangzhou, Guangdong [16]	
≥25years‡	Female 27.0, male 33.9
Yutian of Shanxi [16]	
≥25years‡	Female 56.1, male 66.1
Kelamayi of Xinjiang [16]	
≥25years‡	Female 29.5, male 44.4

* Age-adjusted rates, calculated according to the European standard population.

¶ Age-adjusted rates, calculated according to the United States of American standard population in 1970.

‡ Age-adjusted rates, computed by the direct method with the Chinese population age 25 years and older in 2000 as the standard.

† Depending on vaccine.

§ Data from passive surveillance.

Unpredictable pathogens may cause increased rates of spontaneous abortion and premature labour. Table 2 shows the rates of spontaneous abortion among 31 provinces of mainland, China [17]. Spontaneous abortion rates were the highest in Shanghai (12.70%) and the lowest in Jilin (0.84%), and varied from 2.70% to 12.20% in other provinces. Rates of preterm labour or delivery were similar among provinces, varying from 4.7% to 7.8% (Table 3) [18]–[23].

**Table 2:** Spontaneous abortion rates (SAR) by province from 1988 to1997 in China [17].

Province	SAR (%)	Province	SAR (%)	Province	SAR (%)
Beijing	8.70	Anhui	3.37	Guizhou	2.80
Tianjin	2.70	Fujian	3.27	Yunnan	4.68
Hebei	4.50	Jiangxi	4.50	Tibet	8.77
Shanxi	6.49	Shandong	3.22	Chongxing	4.19
Inner Mongolia	6.58	Henna	1.99	Shaanxi	4.94
Liaoning	6.69	Hubei	4.55	Gansu	3.55
Jilin	0.84	Hunan	4.94	Qinghai	10.00
Heilongjiang	5.29	Guangdong	2.54	Ningxia	12.20
Shanghai	12.70	Guangxi	3.39	Uyghur	3.77
Jiangsu	4.79	Hainan	1.49		
Zhejiang	5.26	Sichuan	4.27		

**Table 3:** Rates of preterm labour or delivery (<37 weeks gestation) by provinces in China.

Provinces in China	Rates (%)
Beijing (2006–2007) [18]	6.3
Hong Kong (1995–1996) [19]	7.4
Guangzhou in Guangdong (1969–1998) [20]	4.7
Gansu (1992–2001) [21]	6.4
Hunan (1992–2001) [22]	7.8
Bengbu, Anhui [23]	5.9

Table 4 shows the number of coincident events that might be expected as background rate events within one day, one week, and six weeks of receiving a dose of hypothesized vaccine, in China. Even for rare events, a large number of events can be expected in a short time period because of the large population targeted for immunization [10], [15]–[18]. For example, if a cohort of 10 million individuals were vaccinated, 7.71 cases of Guillain-Barré syndrome would be expected to occur within six weeks of vaccination as coincident background cases. If 1 million migrant workers were vaccinated, 126.58 cases of sudden adult death syndrome would be expected to occur within one day of vaccination as coincident background cases. For some of the other events, such as preterm labour, the numbers of expected events are quite large. Among 1 million vaccinated pregnant women with a stillbirth of 196–258 days, 1,381 preterm labours are predicted to occur within one day after the vaccination.

**Table 4:** The predicted numbers of coincident temporarily associated events after immunization.

	Within 1 day	Within 7 days	Within 6weeks	Baseline rate used for estimate
Guillain-Barré syndrome (per 10 million vaccinated people)	0.28	1.96	11.74	1.02 per 100, 000 person-years (all ages, male, urban of Beijing) [10]
Sudden adult death syndrome (per 1 million vaccinated migrant workers)	3.01	21.10	126.58	110 per 100, 000 person-years (15∼52 years, migrant workers of Guangdong) [15]
Sudden cardiac death (per 1 million vaccinated people)	1.02	7.15	42.92	37.3 per 100, 000 person-years (≥25 years, male, Beijing) [16]
Preterm labour (per 1 million vaccinated pregnant women with a stillbirth of 196–258 days)	1381	9667	58000	Based on background rate of 8.7% pregnancies with a stillbirth of 196–258 days in Beijing [17]
Spontaneous abortion (per 1 million vaccinated pregnant women with a stillbirth of less than 195 days)	323	2262	13569	Based on background rate of 6.3% pregnancies with a stillbirth of less than 195 days in Beijing [18]

## Discussion

Mass immunization will magnify the adverse events of vaccine, and poor public understanding of these events will lead to misinterpreting coincidence for causal association. For example, the seasonal influenza vaccine campaign in 2006, Israel, was interrupted because four deaths occurred within 24 h of immunization. These four deaths were then proved to be coincident sudden deaths because the four patients were at high risk of sudden death due to their ages and underlying disorders. The vaccine campaign restarted after the clarification [24].

Randomized, placebo-controlled trials might be the best method in monitoring vaccine safety; however, large amount of samples are needed, especially to monitor events with a very low incidence rate [25]. For example, the incidence of Guillain-Barré syndrome within seven days is 1.96 syndromes per 10 million vaccinated people. Supposing the incidence increases 1,000 times (α = 0.05 and β = 0.10), the sample size would be 53,782 subjects for both groups. Even the large trials carried out in the United States (1,323 participants), Hungary (368 participants) and China (12,691 participants) do not reach the power to screen adverse events with such low incidence rates, but strongly associated with vaccination [26]–[28].

The Emergency Response Office of the Ministry of Health in China reported at a Press Conference in Beijing on the December 1, 2010 that 2,867 suspected adverse reactions had occurred after receipt of the China-made A/H1N1 flu vaccine. In addition to this, four deaths were also reported; three were coincidental and the other yet to be determined [1]. Although the exact number would vary with the demographics of the vaccinated population, the predicted incident rates of background events could be a simple and quick toll to evaluate the safety of a vaccine. Of 10 million vaccinated people in China, 11.46 cases of sudden cardiac death are predicted to occur within one day of immunization. Judging by this prediction, the event of four deaths would be easily explained as coincident sudden deaths, i.e., China-made A/H1N1 flu vaccine is safe, by reference to the sudden cardiac death rate.

China, with a land of 9.6 million square kilometers and a population of 1.3 billion, consists of 56 ethnic groups with multiple lifestyle and language backgrounds. These different cultural, living environment, lifestyle and economic development levels contribute to a wide variation of incidence rates in epidemic events. For example, the spontaneous abortion rates (SAR) by province ranged from 0.84% (Jilin) to 12.70% (Shanghai), and such variation might come from lifestyle habits, economic and health condition, climate, and different marriage patterns, especially “age at first marriage” and “total number of pregnancies” [17]. Therefore, it is very complex to distinguish events temporarily associated with, but not caused by, vaccination from those due to vaccination itself. For these reasons, we try to summarize those events from as many regions in China as possible to cover the general population instead of a systematic review or a meta-analysis on some specific events from a special geographical area or a special ethnic group.

In addition to the importance of background rates of events in assessment of vaccine safety during mass immunization, the background rates could also play important roles in vaccine education. In a survey preformed in medical students, we found that awareness of the background rate of sudden cardiac death increases the intended vaccination rate of pandemic H1N1 influenza vaccines, from 30.30% to 48.15% [29].

Recently emerging infectious diseases, such as SARS in 2003, H5N1 influenza in 2005, pandemic H1N1 influenza in 2009, and H7N9 influenza in 2013, seriously threaten human health. Our findings supply the expected number of background disease events during mass immunization against emerging infectious disease, and this is the first attempt in China to estimate the safety of mass immunization with the available data. The result could also act as the reference for screening the safety of vaccines against unpredictable pathogens in the future.

## References

[1] Ministry of Health, People's Republic of China. (2010) 5th Health Bulletin of 2010. Ministry of Health Report, 2 March, 2010. Available: http://www.moh.gov.cn/publicfiles/business/htmlfiles/mohwsyjbgs/s7863/201003/46150.htm. Accessed 2010 Mar 2. (in Chinese).

[2] Black S, Eskola J, Siegrist CA, Halsey N, Macdonald N, et al (2009) Importance of background rates of disease in assessment of vaccine safety during mass immunisation with pandemic H1N1 influenza vaccines. Lancet 374: 2115–2122. 19880172 10.1016/S0140-6736(09)61877-8 PMC2861912

[3] Deeks SL, Lim GH, Simpson MA, Rosella L, Mackie CO, et al (2011) Estimating background rates of Guillain-Barré Syndrome in Ontario in order to respond to safety concerns during pandemic H1N1/09 immunization campaign. BMC Public Health 11: 329. 21586163 10.1186/1471-2458-11-329 PMC3112136

[4] China National Knowledge Infrastructure. Available: http://www.global.cnki.net. Accessed 2009 Oct 1.

[5] Wanfang Data. Available: http://www.wanfangdata.com. Accessed 2009 Oct 1. Chinese Medical Current Content. Available: http://www.cmcc.org.cn. Accessed 2009 Oct 1.

[6] Rudan I, Chan KY, Zhang JS, Theodoratou E, Feng XL, et al (2010) WHO/UNICEF's Child Health Epidemiology Reference Group (CHERG). Causes of deaths in children younger than 5 years in China in 2008. Lancet 375: 1083–1089. 20346815 10.1016/S0140-6736(10)60060-8

[7] Xia J, Wright J, Adams CE (2008) Five large Chinese biomedical bibliographic databases: accessibility and coverage. Health Info Libr J 25: 55–61. 18251914 10.1111/j.1471-1842.2007.00734.x

[8] Fung ICH (2008) Chinese journals: a guide for epidemiologists. Emerg Themes Epidemiol 5: 20. 18826604 10.1186/1742-7622-5-20 PMC2648956

[9] Cheng Q, Wang DS, Jiang GX, Han H, Zhang Y, et al (2002) Distinct pattern of age-specific incidence of Guillain-Barré syndrome in Harbin, China. J Neurology 249: 25–32. 10.1007/pl00007844 11954865

[10] Hong X, Zhang Z, Wang J, Zhang B, Yin W, et al (2000) Epidemiological Characteritics of Guillain-Barré syndrome in urban and rural areas in Beijing and Hebei, China. Acta Academiae Medicinae Sinicae 22: 115–119. 12903510

[11] Shu M, Liu Q, Wang J, Ao R, Yang C, et al (2011) Measles vaccine adverse events reported in the mass vaccination campaign of Sichuan province, China from 2007 to 2008. Vaccine 29: 3507–3510. 19909830 10.1016/j.vaccine.2009.10.106

[12] Wang W, Wu J, Wang D, Chen G, Wang T, et al (2002) Epidemiological survey on epilepsy among rural populations in five provinces in China. Natl Med J China (Chin) 82: 49–52. 12133512

[13] Ren H, Yu ZP, Wang WM, Gang LZ, Li YL (2007) Epidemiological survey on epilepsy in Jinuo ethnic minority group in Yunnan Province. J Int Neurology Neurosurgery 34: 10–12.

[14] Da GW (1997) Epidemiological survey on epilepsy in 30,000 Tibetans farmers in Lhase. Tibetan J Med (Chin) 18: 23–24.

[15] Cheng JD, Li HX, Li J, Lu CY, Tang SB, et al (2008) The current epidemic circumstance of sudden unexplained nocturnal death syndrome in Chinese-The epidemiological study of sudden unexplained nocturnal death syndrome in Dongguan, Longgang and Baoan District. Int J Intern Med 125: 21–28.

[16] Hua W, Zhang LF, Wu YF, Liu XQ, Guo DS, et al (2009) Incidence of sudden cardiac death in China: analysis of 4 regional populations. J Am Coll Cardiol 54: 1110–1118. 19744622 10.1016/j.jacc.2009.06.016

[17] Liu B, Gao ES (2002) Risk Factors for Spontaneous Abortion of Chinese Married Women at Reproductive Age. Chn Public Health 18: 890–892.

[18] Guo ZK, Ma JM, Fan L, Zhang YP, Yang Z, et al (2010) Preterm birth and preterm infants in Beijing regional district. Chn J Obstet Gynecol (Chin) 45: 99–103. 20420778

[19] Leung TN, Roach VJ, Lau TK (1998) Incidence of preterm delivery in Hong Kong Chinese. Aust NZ J Obstet Gynecol 38: 138–141. 10.1111/j.1479-828x.1998.tb02986.x 9653845

[20] Chen ML, Huang SY, Wang ZL (2001) The morbidity trend of the preterm delivery in thirty years and sampling analysis of the obstetrical factors. Academic J Sun Yat-Sen Univ. Medical Sci (Chin) 22: 68–72.

[21] Luo YL, Fei CY, Che Y, Zhou WJ (2007) Studies on Premature Delivery and Its Affecting Factors-Analyses of Delivery from Obsterics Department of Gansu Provincial Hospital. J Gansu Sci (Chin) 19: 81–83.

[22] Wu ZD, Fu D, Wu XH, Zhang WS (2005) Clinical analysis of 554 cases of preterm labour. J Chn Physician (Chin) 7: 670–671.

[23] Yao RY, Han H, Zhang YY (2005) The survey analysis of risk factors for preterm delivery. J Bengbu Med College (Chin) 30: 500–502.

[24] Kokia ES, Silverman BG, Green M, Kedem H, Guindy M, et al (2007) Deaths following influenza vaccination-background mortality or causal connection? Vaccine 25: 8557–8561. 18006121 10.1016/j.vaccine.2007.10.021

[25] Black N (1996) Why we need observational studies to evaluate the effectiveness of health care. Brit Med J 312: 1215. 8634569 10.1136/bmj.312.7040.1215 PMC2350940

[26] Plennevaux E, Sheldon E, Blatter M, Reeves-Hoché MK, Denis M (2010) Immune response after a single vaccination against 2009 influenza A H1N1 in USA: a preliminary report of two randomized controlled phase 2 trials. Lancet 375: 41–48. 20018365 10.1016/S0140-6736(09)62026-2

[27] Vajo Z, Tamas F, Sinka L, Jankovics I (2010) Safety and immunogenicity of a 2009 pandemic influenza A H1N1 vaccine when administered alone or simultaneously with seasonal influenza vaccine for the 2009–10 influenza season: a multicentre, randomized controlled trial. Lancet 375: 49–55. 20018367 10.1016/S0140-6736(09)62039-0

[28] Liang XF, Wang HQ, Wang JZ, Fang HH, Wu J, et al (2010) Safety and immunogenicity of 2009 pandemic influenza A H1N1 vaccines in China: a multicentre, double-blind, randomized, placebo-controlled trial. Lancet 375: 56–66. 20018364 10.1016/S0140-6736(09)62003-1

[29] Wang Y, Xiao Z, Wang W (2010) Awareness of the background rate of sudden cardiac death during mass immunization with pandemic H1N1 influenza vaccines increases the intended vaccination rate. Prev Med 51: 445–446. 20832419 10.1016/j.ypmed.2010.08.019

